# Complete genome sequence of *Moraxella* sp. strain DOX410, a bacterium potentially degrading 1,4-dioxane isolated from river sediment

**DOI:** 10.1128/mra.00933-23

**Published:** 2023-12-06

**Authors:** Byung-Gon Ryu, Hye Kyeong Kang, Mi-Hwa Lee, Byung Hee Chun

**Affiliations:** 1 Environmental Microbiology Research Team, Microbial Research Department, Nakdonggang National Institute of Biological Resources, Sangju-si, South Korea; 2 Department of Microbiology, Pukyong University, Busan, South Korea; DOE Joint Genome Institute, Berkeley, California, USA

**Keywords:** *Moraxella*, complete genome, 1,4-dioxane-degrading bacterium

## Abstract

*Moraxella* sp. strain DOX410 was isolated from a systematic enrichment culture to investigate strains with 1,4-dioxane degradation potential. The genome of strain DOX410 consists of five complete contigs and contained genes related to degradation of the aromatic ring structure of 1,4-dioxane.

## ANNOUNCEMENT

1,4-Dioxane, a compound classified as a Group 2B carcinogen, is a significant environmental concern due to its persistent presence in surface waters and frequently detected at elevated levels, posing risks to both aquatic life and potentially to human health (1). As a result, investigating microbes capable of 1,4-dioxane degradation, along with the elucidation of their genetic pathways, represents a critical step towards devising effective strategies for environmental remediation and ensuring water quality (2).

To isolate the 1,4-dioxane-degrading bacteria, approximately 10 g of sample collected from river sediment (36°05′22.5″N 128°22′14.3″E) was incubated in R2A broth supplemented with 200 ppm of 1,4-dioxane at 28°C. The enrichment culture was transferred (1:10) three times into fresh R2A broth containing 200 ppm of 1,4-dioxane every 2 weeks. After systematic enrichment culture, a colony named DOX410 showing potential 1,4-dioxane-degrading activity was isolated from enrichment culture and transferred to R2A agar for pure culture. The colony of strain DOX410 was cultured in R2A broth and stored at −80°C in R2A broth containing 15% (vol/vol) glycerol for long-term preservation and genome extraction. The 16S rRNA gene of strain DOX410 was PCR amplified with 27F and 1492R primer (3). The PCR product was sequenced by Sanger sequencing, and the resulting 16S rRNA gene sequence was compared with those of validated type strains in the EzTaxon-e server (4). Strain DOX410 was most closely related to *Moraxella osloensis* CCUG 350^T^ with a similarity of 98.9%. For genomic DNA extraction, strain DOX410 was cultured in R2A broth at 28°C for 3 days and the genomic DNA from cultured cells was extracted using TaKaRa MiniBEST Bacteria Genomic DNA Extraction Kit Ver.3.0 according to manufacturer’s instruction. An extracted genomic DNA was sequenced by a PacBio Sequel II system to produce complete genome sequences and an Illumina HiSeq 4000 platform for error correction at Microgen (Korea). The libraries were constructed using the SMRTbell Express Template Prep Kit for PacBio sequencing and Illumina NGS library prep kit for Illumina sequencing. A total of 19,605,884 high-quality reads with 2,959,533,725 read bases (N50, 8,976 bp), 43.98% of GC content, and 93.38% of Q30 were generated after quality control of HiFi sequencing result using SMRTlink software version 11.1 (5). Through *de novo* assembly using Microbial Genome Assembly application available in SMRTlink and error correction based on Illumina reads using Pilon version 1.21 (6), five complete circular contigs consisting of a total length of 2,600,244 bp with 190.7 × coverage were produced (Table 1). The quality analysis using CheckM (7) showed 95.83% for completeness and 6.19% for contamination rate. The genome annotation with NCBI Prokaryotic Genome Annotation Pipeline v6.6 (8) identified 2,356 total genes (coding sequence, CDS), 2,293 protein-coding genes, 12 rRNA genes, 47 tRNAs, 4 ncRNAs, and 41 pseudogenes.

**TABLE 1:** Genomic features of five contigs in the strain DOX410 genome

Genomic features	Contigs				
	1	2	3	4	5
Length (bp)	2,399,733	70,594	62,413	56,226	11,278
GC content (%)	44.0	40.6	41.5	39.1	37.2
Coverage (**×**)	184.4	172.3	312.3	195.5	957.2
GenBank accession no.	CP135138	CP135139	CP135140	CP135141	CP135142

KEGG database (9) and BLASTP searches identified genes related to producing class III extradiol ring-cleavage dioxygenase (RNZ41_00620 and RNZ41_01005) and carboxymuconolactone decarboxylase (RNZ41_04330), which can be involved in the degradation of the aromatic ring structure of 1,4-dioxane. The genome would be helpful for understanding the mechanism of 1,4-dioxane degradation and for further bioinformatic studies.

## Data Availability

The complete genome sequencing data of strain DOX410 derived in this study are publicly available in the GenBank database under accession number CP135138-CP135142 (NCBI BioProject number: PRJNA1019065). The raw reads for PacBio and Illumina sequencing are publicly available in the Sequence Read Archive under accession numbers SRR26553382 and SRR26553383, respectively.
